# How Good Are Indirect Tests at Detecting Recombination in Human mtDNA?

**DOI:** 10.1534/g3.113.006510

**Published:** 2013-07-01

**Authors:** Daniel James White, David Bryant, Neil John Gemmell

**Affiliations:** * Biodiversity and Informatics, Landcare Research New Zealand, Auckland, 1072; † Department of Mathematics and Statistics, University of Otago, Dunedin, 9054, New Zealand; ‡ Allan Wilson Centre for Molecular Ecology and Evolution, University of Otago, Dunedin, 9054, New Zealand; § Centre for Reproduction and Genomics, Department of Anatomy, University of Otago, Dunedin, 9054, New Zealand

**Keywords:** recombination, mtDNA, indirect tests, effectiveness, human

## Abstract

Empirical proof of human mitochondrial DNA (mtDNA) recombination in somatic tissues was obtained in 2004; however, a lack of irrefutable evidence exists for recombination in human mtDNA at the population level. Our inability to demonstrate convincingly a signal of recombination in population data sets of human mtDNA sequence may be due, in part, to the ineffectiveness of current indirect tests. Previously, we tested some well-established indirect tests of recombination (linkage disequilibrium *vs.* distance using D′ and r^2^, Homoplasy Test, Pairwise Homoplasy Index, Neighborhood Similarity Score, and Max χ^2^) on sequence data derived from the only empirically confirmed case of human mtDNA recombination thus far and demonstrated that some methods were unable to detect recombination. Here, we assess the performance of these six well-established tests and explore what characteristics specific to human mtDNA sequence may affect their efficacy by simulating sequence under various parameters with levels of recombination (ρ) that vary around an empirically derived estimate for human mtDNA (population parameter ρ = 5.492). No test performed infallibly under any of our scenarios, and error rates varied across tests, whereas detection rates increased substantially with ρ values > 5.492. Under a model of evolution that incorporates parameters specific to human mtDNA, including rate heterogeneity, population expansion, and ρ = 5.492, successful detection rates are limited to a range of 7−70% across tests with an acceptable level of false-positive results: the neighborhood similarity score incompatibility test performed best overall under these parameters. Population growth seems to have the greatest impact on recombination detection probabilities across all models tested, likely due to its impact on sequence diversity. The implications of our findings on our current understanding of mtDNA recombination in humans are discussed.

If recombination is occurring in human mitochondrial DNA (mtDNA) at a sufficiently high level, then many evolutionary analyses based on the molecule (Anisimova *et al.* 2003; Arenas and Posada 2010a,b; Schierup and Hein 2000a,b), and indeed the models of evolution of the molecule itself, will need to undergo substantial revision. The question of whether recombination occurs at this level is, however, largely disregarded by the scientific community. This consensus has been reached based on a number of factors. It was long held that the mtDNA molecule evolves in a strictly clonal manner, with exclusively uniparental inheritance and no recombination. In fact, originally, it was not clear whether enzymes required for recombination were present in mitochondria at all. Furthermore, it was accepted that significant-enough mitochondrial heteroplasmy did not exist (more than one mitotype present in a cell or individual) for there to be any biological impact even if recombination were to occur. Early studies in which investigators reported apparent signals of recombination in human mtDNA (Awadalla *et al.* 1999; Eyre-Walker *et al.* 1999; Hagelberg *et al.* 1999) were largely discounted by statistical arguments (Innan and Nordborg 2002; Jorde and Bamshad 2000; Kumar *et al.* 2000) and inaccuracies in sequence data (Hagelberg *et al.* 2000; Kivisild and Villems 2000).

However, the body of evidence supporting the potential for biologically important recombination in human mtDNA has increased over recent years. For example, in 1999 it was discovered that the enzymatic machinery necessary for recombination is present in mitochondria (Lakshmipathy and Campbell 1999; Thyagarajan *et al.* 1996). Important revelations came in 2004 and 2005, when widespread mtDNA recombination was discovered in up to 14.2% and 16.1% of animal populations, respectfully (Piganeau *et al.* 2004; Tsaousis *et al.* 2005), although a recent and interesting study has shown that positive results for recombination in animal mtDNA could be an artifact of a specific form of mutation rate heterogeneity (Sun *et al.* 2011). Experimental work has shown that recombination can occur between heterologous mitochondrial molecules, harboring pathogenic mutations, in human cytoplasmic hybrid cells (D’Aurelio *et al.* 2004). Finally, studies on the patterns of tetraplasmy in doubly heteroplasmic individuals not only suggest mitochondrial recombination to be relatively common in human skeletal muscle tissue but also demonstrate the inheritability of recombinant mtDNA molecules (Zsurka *et al.* 2005, 2007).

For recombination to have a biological impact, it must occur between two different mitotypes; thus, a state of mitochondrial heteroplasmy is required. To this end, it is now accepted that mitochondrial heteroplasmy is a regular feature among higher eukaryotes (White *et al.* 2008). As evidence, the paternal inheritance of mtDNA, a common source of heteroplasmy, is relatively widespread across the animal kingdom (Fontaine *et al.* 2007; Gyllensten *et al.* 1991; Sherengul *et al.* 2006; Zhao *et al.* 2004), and is also known to occur in humans (Schwartz and Vissing 2002; Zsurka *et al.* 2005). Indeed, human mitochondrial heteroplasmy can persist in the germline over several generations (Ivanov *et al.* 1996).

The most convincing *direct* evidence of a recombination event in human mtDNA to date remains that of a male sufferer of a mitochondrial myopathy, who had a 10:1 ratio of paternal to maternal mtDNA in his skeletal muscle. A deletion in his paternally derived mtDNA gave rise to the disorder, and it was found that 0.7% of mtDNA molecules were recombinant (Kraytsberg *et al.* 2004). This research group subsequently went on to show that recombination in this tissue might be relatively common (Zsurka *et al.* 2005).

To understand the appropriate role of recombination in evolutionary analyses centered on the mtDNA molecule, we require more than sporadic cases leading to dysfunction. Instead, we require evidence for mitochondrial recombination at a population level. Such evidence can be provided by the numerous statistical tests that estimate the probability that polymorphism distributions in DNA sequence are due to recombination (Bruen *et al.* 2006; Posada and Crandall 2001, see http://www.bioinf.manchester.ac.uk/recombination/programs.shtml for an up to date list). The early evidence for mitochondrial recombination in human populations using these methods (Awadalla *et al.* 1999; Eyre-Walker *et al.* 1999) is considered equivocal at best, however, and has largely been disregarded. The reasons for refuting the positive signals for recombination as revealed in these studies include the incorrect choice of statistical test (Jorde and Bamshad 2000; Kumar *et al.* 2000), the quality of sequence data (Kivisild and Villems 2000), and inaccurate interpretation of results (Innan and Nordborg 2002). Subsequent attempts to repeat the findings of the first studies have been unsuccessful (Elson *et al.* 2001; Piganeau and Eyre-Walker 2004).

The question we focus on here is why there is such a lack of compelling evidence for mtDNA recombination frequency in human populations, given that there is definitely potential for biologically relevant mitochondrial recombination to occur, and it is occurring at the population level in our animal counterparts. With a lack of compelling evidence, the majority of the scientific community is content to accept that it may simply just not be occurring at a significantly high enough level for due concern.

One intriguing possibility is that recombination may well be occurring in human mtDNA but that we are not actually able to detect it with the current set of tools. In a previous study, we demonstrated that some of the most robust indirect statistical tests for recombination detection are not effective at detecting recombination in human mtDNA, even when it is known to have occurred (White and Gemmell 2009).

Here, we explore how some of the processes under which human mtDNA evolves may affect the effectiveness of indirect tests of recombination detection. Specifically, we evolve DNA sequence under parameters consistent with human mtDNA, including rate heterogeneity and population expansion. We perform these simulations by using variable levels of recombination to determine the limitations of six well-established indirect tests of recombination. The power of these recombination tests has been comprehensively assessed elsewhere (Bruen *et al.* 2006; Posada and Crandall 2001; Wiuf *et al.* 2001) and our objective here is not to repeat such work. Rather, our intent is to assess the effects, on the detection abilities of popular tests of recombination, of some of the key parameters known to impact the evolution of this molecule in humans. In particular, we ask the question, what level of recombination in human mtDNA might we need before it can be reliably detected with the currently available recombination detection tools? We find that current indirect tests of recombination perform very poorly even under our simple models of sequence evolution, and likely will perform even worse as model complexity increases toward a scenario closer to biological reality. We discuss how our findings could influence previous studies, and current opinions, of human mtDNA recombination.

## Materials and Methods

### Sequence simulation

Sequence was simulated under a relatively simple scenario consistent with the evolution of human mtDNA, with varying levels of recombination. To estimate the population parameters to use for the simulation of sequences, recombinant mtDNA sequences from the only known case of empirical recombination detection in human mtDNA (Kraytsberg *et al.* 2004) were run in LAMARC v2.1 (Kuhner 2006). Assuming a mutation rate, μ, of 5 × 10^−7^ per site per generation for the mtDNA molecule (Mishmar *et al.* 2003), the required number of individuals to generate the observed genetic diversity within this sample of recombined sequences, effective population size N, was approximated at 1370 (from N = θ/μ, where θ is the mutation rate parameter). The recombination factor, R, was estimated in LAMARC from R = r/μ, where r is the recombination rate per intersite link per generation and μ is the mutation rate per site per generation (Kuhner 2006) and can be described as the relative frequency of recombination next to a given site compared with a mutation at that site. For our sequences, and assuming μ to be 5 × 10^−7^, R was estimated at 1.170 giving an r value of 5.8485 × 10^−7^ (see White and Gemmell 2009 for more details).

These parameters were incorporated into RECODON v1.5.0, which generates sequence under a coalescent model (Arenas and Posada 2007). Population parameters per locus were estimated from a product of twice the number of inheritable copies, the per-site factor and the length in basepairs of the locus, l. As such, the per-locus population recombination parameter, ρ, was approximately Nrl for mitochondrial sequence when we assume an equal number of females and males in the population (Arenas and Posada 2007); we set l to 6854 bp, which gives a value for ρ of 5.492 for the empirical data set. To account for recombination occurring only in females for mtDNA, the per-site recombination rate was halved for the simulations; otherwise, the level of recombination is overestimated in the simulated sequences. Three models of sequence evolution were used: (1) constant population size and homogeneous mutation rate; (2) constant population size and heterogeneous mutation rate; and (3) population growth and heterogeneous mutation rate. For models that included rate heterogeneity a gamma distribution of mutation rates with alpha set at 0.2 was used (Yang 1996), and for the model that included population growth an exponential growth rate of 1 × 10^−3^ per generation was incorporated (Pluzhnikov *et al.* 2002). For each evolutionary model, ρ was set to 0, 2, 5.492, 8, 10, and 12 to span the estimated value for mtDNA (5.492), and 100 samples of 33 sequences were generated for each level of recombination. Sequences were evolved under the Jukes-Cantor nucleotide model assuming equal frequencies for all nucleotides, with an equal transition-transversion ratio.

### The tests

Six well-known indirect tests of recombination were chosen for assessment. These include three population-based tests: the correlation between linkage disequilibrium (LD) and physical distance using the D− measure of LD; the correlation between LD and physical distance using the r^2^ measure of LD; and the Homoplasy Test (Smith and Smith 1998). Three general methods also were assessed, including Max χ^2^ (Smith 1992), the Neighborhood Similarity Score (NSS) (Jakobsen and Easteal 1996), and the more recently developed Pairwise Homoplasy Index (PHI) test (Bruen *et al.* 2006). We have described these indirect tests of recombination in some detail elsewhere (White and Gemmell 2009) but briefly re-encapsulate the key points of difference among them here.

The use of LD to predict recombination relies on a significant negative correlation between physical distance and strength of LD in the presence of recombination (Awadalla *et al.* 1999). For both measures of LD, Pearson’s correlation coefficient was used, and the statistical significance of the correlation was estimated after 1000 random permutations of the data using a Mantel test, all implemented in RecombiTEST (Piganeau *et al.* 2004). The Homoplasy Test is used to examine whether there are more homoplasies on a phylogenetic tree than expected under a model of clonal inheritance, where a homoplasy is the co-occurrence of a polymorphism on separate branches of the tree (Smith and Smith 1998). We implemented this test in the Linux operating system by using a C translation of the original QBasic version, an unsupported version kindly provided by David Posada (University of Vigo). To simulate the process of synonymous site selection, which is a recommended step to control for the compounding effects of selection on recombination detection, a second file was generated for analysis by using every third base pair of the 6854 bp-long simulated sequence. The C version of Homoplasy was validated by comparing its performance to the original QBasic program using five files with no recombination (ρ = 0) and five with an extreme level of recombination (ρ = 15). The results are shown in Supporting Information, Table S1.

The Max χ^2^ test is used to compare the arrangement of segregating sites either side of a putative crossover break point. Both the NSS and PHI tests look at the distribution of incompatible sites, where sites are compatible only if their history includes no recurrent or convergent mutation or recombination. C versions of Max χ^2^, NSS and PHI (http://www.maths.otago.ac.nz/~dbryant/software.html) were run in Linux, and the significance of results estimated by randomly positioning the informative sites 1000 times and determining the proportion of permuted scores that are: (1) below the observed test score, (2) at least as high as the observed test score, and (3) less than or equal to the observed score, respectfully. For all six tests, a significant signal for recombination was assumed for a dataset if a *P* value of 0.05 or less was achieved. For more detail on the tests see White and Gemmell (2009), and File S1. Simulated sequence data is availabe for download from File S2.

## Results

For the Kraytsberg recombinant sequence data, recombination per site per generation was estimated by LAMARC as 5.849 × 10^−7^, which gives a most probable population recombination parameter per gene, ρ, of 5.492. The population mutation parameter, θ (Nµ), was estimated as 6.850 × 10^−4^, which equates to 4.695 per gene.

### Effect of recombination rate

**Figure 1:**
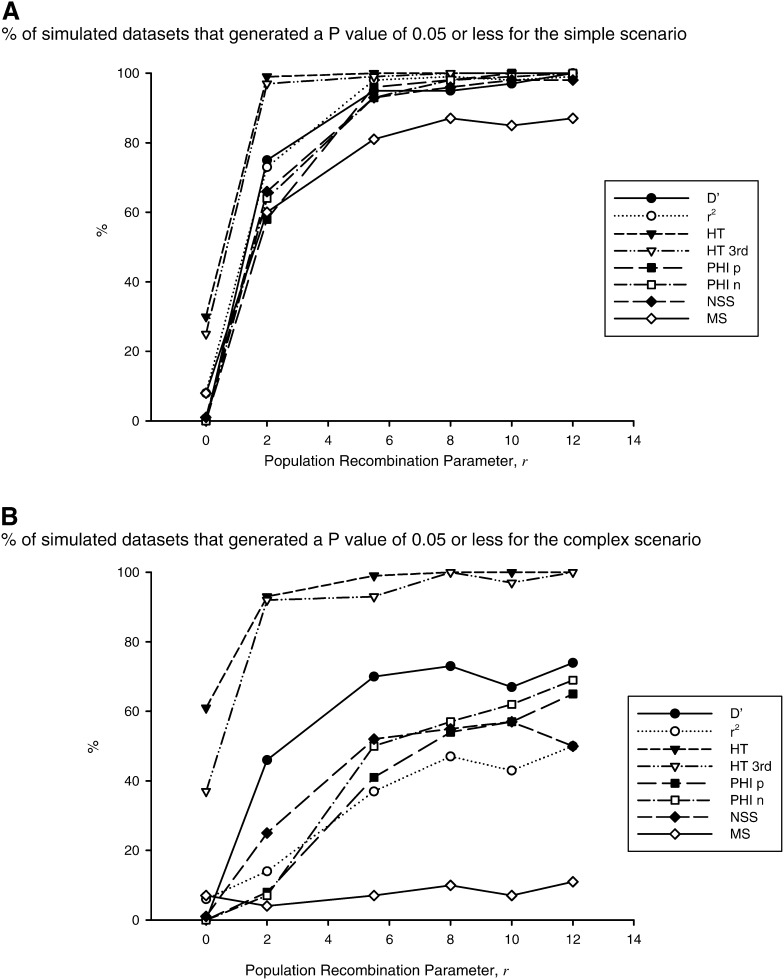
Power of six indirect recombination tests at detecting recombination in simulated mtDNA sequence using (A) a simple mode of evolution and (B) a slightly more complex mode of evolution that incorporates mutation rate heterogeneity and population expansion, over a range of recombination values. D′, LD *vs.* distance using D’; r^2^, LD *vs.* distance using r^2^; HT, Homoplasy Test; HT 3^rd^, Homoplasy Test using only every third site; PHI p, Pairwise Homoplasy Index using permutations; PHI n, Pairwise Homoplasy Index using a normal approximation; NSS, Neighborhood Similarity Score; MS, Max χ^2^.

For sequence simulated under the simplest scenario of no population growth or mutation rate heterogeneity, recombination detection was at, or near, 100% for ρ = 12 for the majority of tests (see Figure 1A). The clear exception to this is the Max χ^2^ test, which reported recombination in only 87% of cases. For the lowest level of recombination with ρ set to 2, only one test (the Homoplasy Test) detected recombination more than 90% of the time. Overall, levels of detection were relatively low for ρ = 2, ranging from 58% to 99%, with a mean of 74.0% (compared with a mean of 98.0% for ρ = 12). Interestingly, for ρ = 5.492 (the value estimated for the empirical example of human mtDNA recombination) the rates of detection ranged from 81 to 100% across all tests, with a mean of 94.4% (Figure 1B).

Although there was a slight reduction in efficiency of recombination detection when only third positions were considered in the Homoplasy Test, compared with when all sites are used, this difference was not significant.

Five of the six tests reported some level of recombination when there was none. Only the PHI test did not. The percentage of false-positive results ranged from 1% for NSS to 30% with the Homoplasy Test when all sites were used.

### Effect of mutation rate heterogeneity

When mutation rate heterogeneity, described by a gamma distribution with α equal to 0.2, is incorporated into sequence evolution, a very similar pattern to the simple scenario is observed in rates of detection across all tests of recombination. When means of detection rates across all tests are compared between the two scenarios, there is no significant difference (*P* < 0.70, paired Student’s *t*-test). When results are compared between the two scenarios for each test separately, Max χ^2^ showed a significant decrease in detection rates at all levels of recombination with mutation rate heterogeneity (*P* < 0.009, paired Student’s *t*-test, Table 1). LD *vs.* distance using r^2^ also showed a smaller, yet significant, reduction in detection rates with mutation rate heterogeneity (*P* < 0.042, paired Student’s *t*-test, Table 1).

**Table 1:** Comparison of recombination detection rates for mtDNA between a simple model of evolution and a model that includes mutation rate heterogeneity but no population growth

Test	Evolutionary Model	Recombination Parameter *ρ*						*P* Value
		0	2	5.492	8	10	12	
D′	Simple	0	75	95	95	97	100	
	rh	5	75	96	88	94	97	0.519
r^2^	Simple	8	73	98	99	98	99	
	rh	3	71	94	99	97	98	0.041
HT	Simple	30	99	100	100	100	100	
	rh	42	96	100	100	100	100	0.518
HT 3^rd^	Simple	25	97	99	100	100	100	
	rh	39	95	99	100	100	100	0.447
PHI p	Simple	0	58	96	98	100	100	
	rh	1	61	95	98	98	100	0.822
PHI n	Simple	0	64	93	98	99	100	
	rh	1	60	96	97	100	99	0.872
NSS	Simple	1	66	93	96	98	98	
	rh	3	71	92	96	97	98	0.419
MS	Simple	8	60	81	87	85	87	
	rh	3	58	77	81	84	85	0.009

Values represent the number of 100 samples in which recombination was detected, using a nominal *P* value of 0.05 to indicate significance. *P* values are from paired, two-tailed Student’s t-tests. Simple, simple scenario of sequence evolution; rh, evolution of sequences involves mutation rate heterogeneity, but no population growth; D′, LD *vs.* distance using D′; r^2^, LD *vs.* distance using r^2^; HT, Homoplasy Test; HT 3^rd^, Homoplasy Test using only every third site; PHI p, Pairwise Homoplasy Index using permutations; PHI n, Pairwise Homoplasy Index using a normal approximation; NSS, Neighborhood Similarity Score; MS, Max χ^2^.

### Effect of mutation rate heterogeneity and population growth

**Figure 2:**
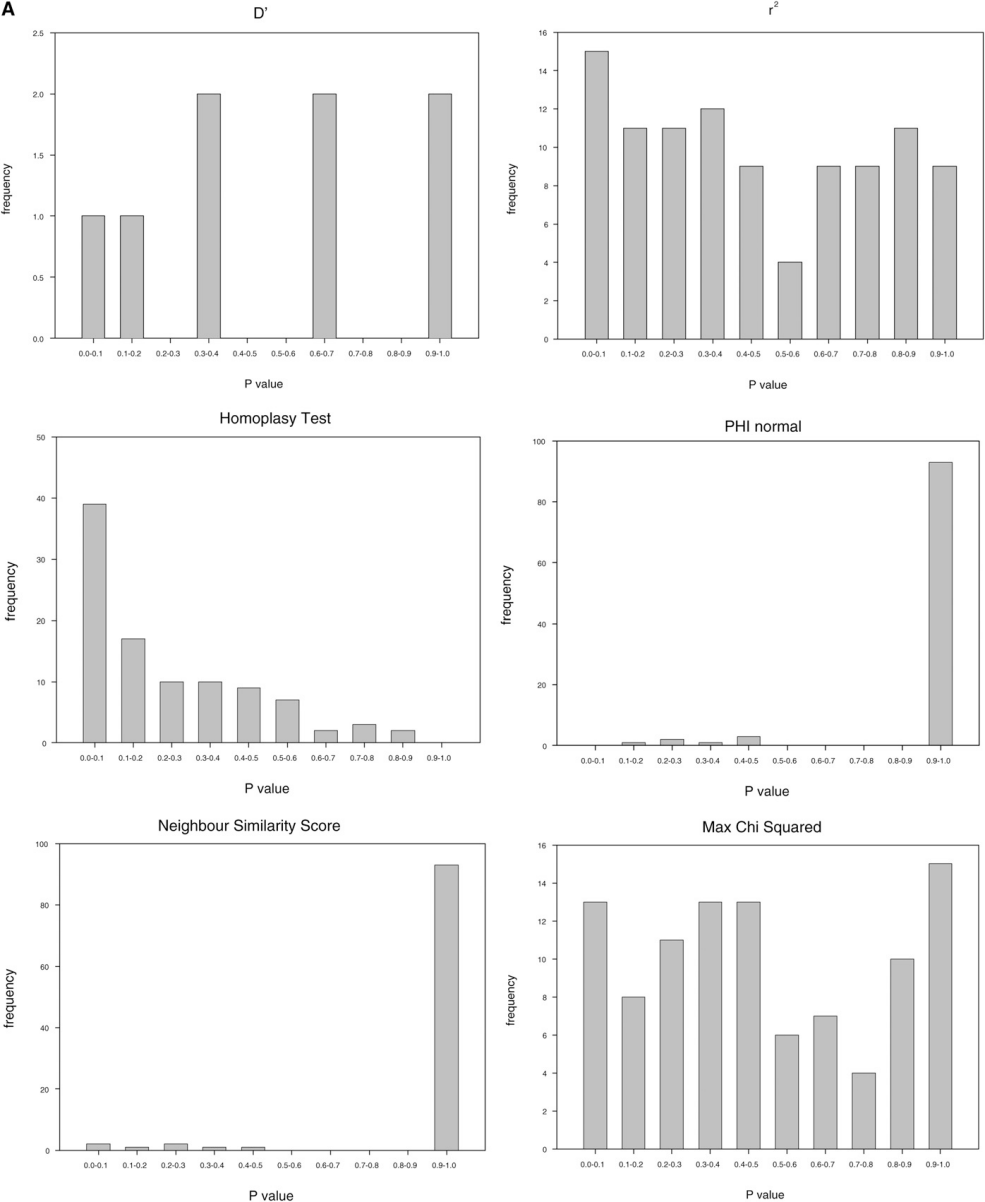

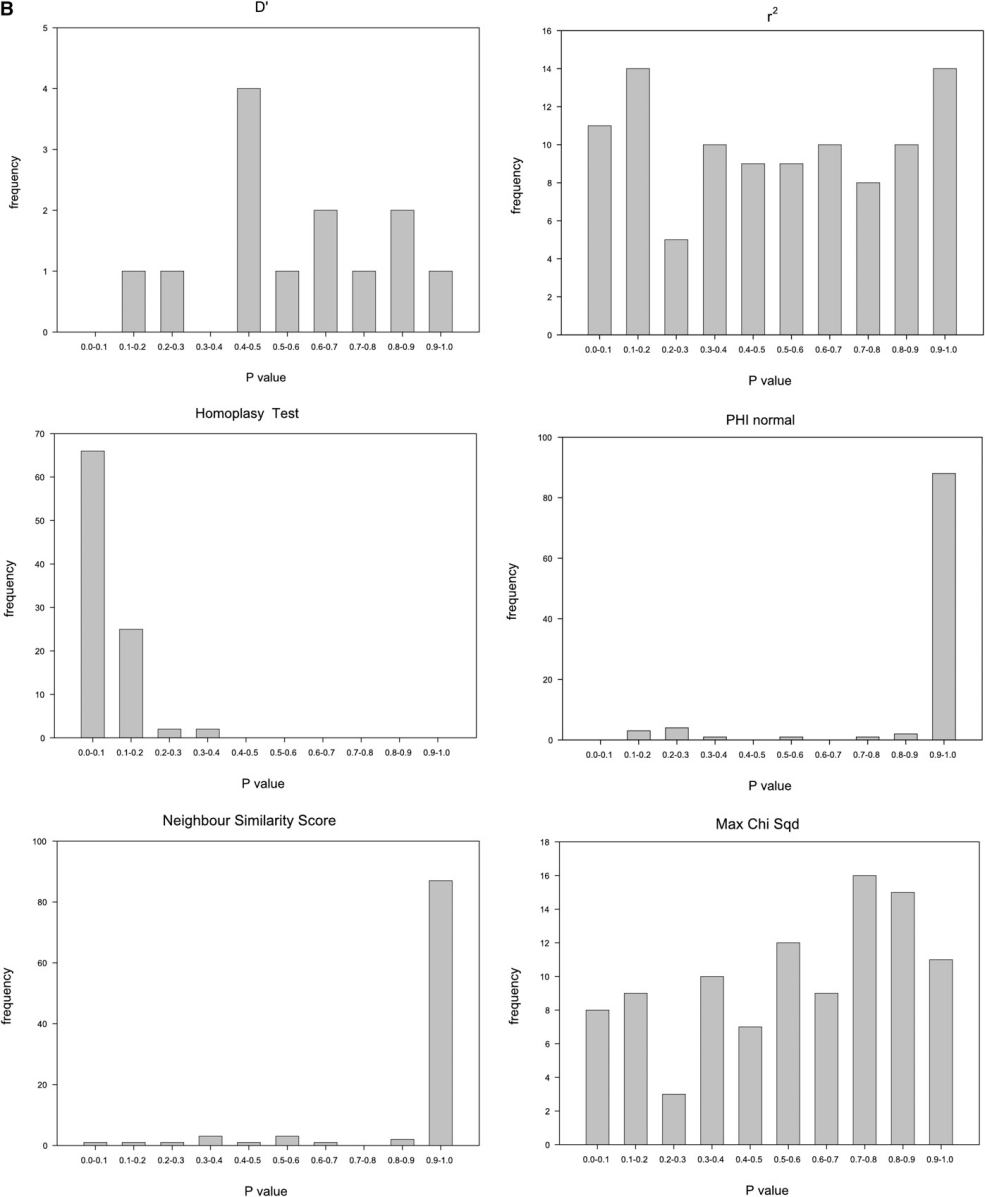
*P* value frequency distribution at *ρ* = 0 across the six indirect tests for the (A) simple model of sequence evolution (no rate heterogeneity or population growth) and (B) slightly more complex model of sequence evolution that incorporates mutation rate heterogeneity and population expansion.

When a population growth rate of 1 × 10^−3^, and mutation rate heterogeneity described by a gamma distribution with α equal to 0.2, were incorporated into simulations, detection rates were significantly lower (*P* < 8.18 × 10^−3^, paired Student’s *t*-test on means for all tests at each value of ρ, see Figure 2). All tests were less able to detect recombination at all levels of recombination. Although the Homoplasy Test maintained the greatest rate of detection, even at low levels of recombination (93% for all sites at ρ = 2), the rate of false-positive results was 61% for this test when all data were examined, and 37% when third positions were used. At the other end of the spectrum, Max χ^2^ performed dismally with detection rates ranging from 4% to 11%. The detection rates of the four remaining tests were similar, ranging from 50% (NSS) to 74% (LD *vs.* distance using D′) at ρ =12. At ρ =5.492, the value estimated for the Kraytsberg recombinant sequence, rates of detection for these four tests ranged from 37% with LD *vs.* distance using r^2^, to 70% with LD *vs.* distance using D′.

A possible reason for the difference in results between simulations without rate heterogeneity and population growth, compared with those that did include these parameters, is the underlying sequence diversity. This has previously been shown to have a strong effect on the efficiency of indirect tests of recombination detection (Bruen *et al.* 2006; Posada and Crandall 2001; Wiuf *et al.* 2001). We calculated the mean nucleotide diversity, π, among sequences (1) in the simple scenario and (2) with mutation rate heterogeneity and population growth as 2.392 × 10^−3^ and 1.257 × 10^−3^, respectively. The latter is comparable with the estimate of π for the empirical data set, 1.250 × 10^−3^ (White and Gemmell 2009). For simulated sequence with mutation rate heterogeneity and no population growth, π among sequences was estimated as 2.545 × 10^−3^.

To assess the impact of the model of sequence evolution on false-positive error rates of the tests, histograms of *P* values obtained for ρ = 0 were plotted for both the most simple model of sequence evolution (Figure 2A) and the model that included population growth and rate heterogeneity (Figure 2B). A uniform distribution of *P* values indicates an unbiased test, a strongly right-skewed distribution indicates a conservative test, whereas a strongly left-skewed distribution indicates high false positive error rates. We observed great variability across tests, with the incompatibility tests (PHI and NSS) having distributions strongly skewed to the right whereas the homoplasy test shows a left skewed distribution.

## Discussion

In this work we have explored the possibility that the lack of evidence for recombination in human mtDNA is not necessarily due to its absence but rather might be a consequence of a lack of power to detect it among current tests. To test this thesis we simulated three sets of sequence with varying levels of recombination: (1) a sequence generated under a simple model using a reliable estimate for the rate of mtDNA mutation (Mishmar *et al.* 2003), but no population growth or mutation rate heterogeneity; (2) a sequence generated using a model that incorporates an unequal distribution of mutation rates across human mtDNA molecules (Yang 1996); and (3) a sequence generated using a model that incorporates a nonuniformly distributed mutation rate, as well as rates of recent human population growth (Pluzhnikov *et al.* 2002).

### Performance of tests

Under the simple model of sequence evolution, the majority of tests were able to detect recombination at least 95% of the time when levels of recombination are relatively high, *i.e.*, with a population recombination parameter, ρ, of at least 8. The exception to this includes the Max χ^2^ test for which a maximum rate of successful detection was estimated at 87%. When ρ is equal to 5.492, the estimated value for human mtDNA based on the Kraytsberg recombinant sequence data (White and Gemmell 2009), detection rates were lower for all tests except the Homoplasy Test and decreased as low as 81% for Max χ^2^ test.

The performance of all tests changed dramatically when population growth and mutation rate heterogeneity were incorporated into the model of sequence evolution, but rate heterogeneity on its own had little effect (Table 1). With the inclusion of an exponential growth factor into sequence simulation, there was a reduction in performance for all tests; most tests showed a marked reduction in their power of detection across all levels of recombination. This effect is most likely explained by the reduction in sequence diversity (*e.g.*, from π = 2.39 × 10^−3^ (SD 0.60 × 10^−3^) in the simple scenario to 1.26 × 10^−3^ (SD 0.41 × 10^−3^)), as the negative impact of low sequence diversity on the tests in this study has been demonstrated before (Bruen *et al.* 2006; Posada and Crandall 2001; Wiuf *et al.* 2001). Most severely affected was the Max χ^2^ test, which is unsurprising given its inefficiency in scenarios of low sequence diversity, particularly under population growth (Bruen *et al.* 2006; Posada and Crandall 2001; Wiuf *et al.* 2001). Thus, this test (using the implementation of Bruen *et al.* 2006) can be excluded as a useful test for detecting recombination under these conditions.

An apparent exception to the overall trend of reduced power under population growth and mutation rate heterogeneity is the Homoplasy Test, which maintained levels of recombination detection greater than 90%, even for a ρ value of 2. The rate of false-positive results, however, more than doubled for this test to 61%. When only third positions are used, a step recommended in real data to prevent the compounding effects of selection on recombination detection (Piganeau and Eyre-Walker 2004; Smith and Smith 1998), the rate of false-positive detection decreases to 37% with the homoplasy test, whereas simultaneously retaining relatively high power.

Of course, a test might have high power not because it is accurate, but because it rejects the null model unnecessarily. We can check for this by simulating with no recombination, and then checking which tests falsely detect recombination. Figure 2 clearly shows that the C version of the Homoplasy Test used here has an unacceptable false-positive rate with our data set, meaning that the apparent low false-negative rate (*i.e.*, high power) of this test is artifactual. In contrast, the incompatibility tests are overly conservative for our sequences, which have similar nucleotide diversity levels to empirical mtDNA data, suggesting inflated false-negative rates with a nominal *P* value cut-off of 0.05 for these tests.

When these results are compared with previous studies (Brown *et al.* 2001; Bruen *et al.* 2006; Posada and Crandall 2001; Wiuf *et al.* 2001) several comparisons can be made. Overall, the apparent power of tests to detect recombination, in a scenario of no population growth, is greater in this analysis than in previous studies (Bruen *et al.* 2006; Posada and Crandall 2001; Wiuf *et al.* 2001). For most tests, recombination is detected at greater percentages across all levels of recombination used here, despite the lower sequence divergence of our simulated sequences. It has previously been shown that the power of these tests increases with increasing sequence diversity (Bruen *et al.* 2006; Posada and Crandall 2001; Wiuf *et al.* 2001); however, we estimated an average pairwise sequence diversity, π, for the simple scenario sequences of 2.392 × 10^−3^ (SD 0.60 × 10^−3^); approximately fivefold less than the least divergent sequences used in previous simulation studies (Bruen *et al.* 2006; Posada and Crandall 2001; Wiuf *et al.* 2001). Reasons for the greater rate of detection here, compared with previous studies, may therefore include a relatively large sample size in this study (n = 33), and a long sequence length (l = 6854 bp), both of which can increase the chance of recombination detection. However, the benefits of a long sequence length may be restricted by the incorporation of a sliding window into sequence analysis (Bruen *et al.* 2006; Wiuf *et al.* 2001).

In their comprehensive review, Posada and Crandall (2001) assessed 14 tests of recombination over a range of parameters and demonstrated that the Homoplasy Test was the most powerful at lower levels of divergence (π ~ 1%). As the level of diversity in our simulated sequences is lower again, and more biologically relevant for studies in humans, an observation of relatively high power with the Homoplasy Test for our data would not be completely unexpected. Posada and Crandall (2001) also showed that, with low mutation rate heterogeneity and low sequence divergence, false-positives should be low with the Homoplasy Test. However, we report a greater false-positive rate than previous studies at 25%, which is substantially larger than expectation (at *P* = 0.05), for the scenario that included no mutation rate heterogeneity.

This observation suggests a discrepancy between the C version of the Homoplasy Test used in the Posada and Crandall’s 2001 study and that used here. To investigate this, we repeated the analyses done in the Posada and Crandall study, which used sequence simulated with lower diversity and shorter length, using our implemented C version. A similar false-positive rate was found as in Posada and Crandall’s study (4%; data not shown). The inflated probability of false-positive results here is therefore due to other factors, such as our larger sequence length and larger effective population size. Larger sequence length could give rise to increased false positives by the method in which the test accounts for homoplasies. With larger sequence length, and corresponding larger number of effective sites, the Homoplasy Test will consider the likelihood that any homoplasies present to have arisen by recurrent mutation to be very small, and instead will report recombination. The type 1 error rate increased to 61% when population growth and rate heterogeneity are included across all sites, and this increase is likely driven, at least in part, by the unequal distribution of mutation rates (Posada and Crandall 2001). The apparent high power with the Homoplasy Test reported here should therefore be treated with caution.

The PHI statistic of Bruen *et al.* (2006) has been reported as a powerful and robust test that can reliably detect recombination under a wide spectrum of parameters, including population growth. Moreover, it has been shown to report few false-positive results, even under strong mutation rate heterogeneity and autocorrelation of substitution rates. However, when there is little sequence diversity, it may be conservative in detecting recombination (Bruen *et al.* 2006). Our results agree with these initial findings in that the power of this test was good at high levels of recombination; however, its performance was reduced when recombination parameters were low. This reduced performance with low recombination is likely due to both reduced sequence diversity, and the incorporation of a sliding window that limits any benefits derived from the long sequence length (6854 bp). NSS, like PHI, is based on incompatibility, and incompatibility methods have been shown to be among the most powerful across a range of parameters (Brown *et al.* 2001; Posada and Crandall 2001; Wiuf *et al.* 2001). Here, it also appears to be successful for mtDNA, under no population growth and a uniform mutation rate. Our results agree with the results of earlier studies in that the incompatibility-based tests (PHI and NSS) are conservative when sequence diversity is low, especially at low levels of recombination (Bruen *et al.* 2006; Posada and Crandall 2001).

The Max χ^2^ method was originally developed for detecting recombination between distinct DNA sequences, showing substantial divergence (Smith 1992). Under such a scenario it has been shown to be a powerful test (Posada and Crandall 2001). Because of the low diversity of our simulated sequences, its poor performance in this study is unsurprising. However, the Max χ^2^ test we used employs the entire sequence and all possible χ^2^ values. If χ^2^ tables with expected values of two or less are excluded, a step that prevents overtly large χ^2^ values being produced when expected values are low, the Max χ^2^ test may become more powerful without inflation of type 1 error (Piganeau and Eyre-Walker 2004).

Historically, there has been some debate as to which measure of LD, r^2^ or D′, is preferable for the detection of recombination. Some authors have questioned the use of LD decay as a reliable indication of recombination at all (Innan and Nordborg 2002). However, the LD association test that we performed compared favorably with four other tests under no population growth in a more recent simulation study in which the authors used both r^2^ and D’ (Bruen *et al.* 2006). Although the first study to show recombination in human mtDNA with LD employed the r^2^ measure (Awadalla *et al.* 1999), it has been argued that only D′, and not r^2^, is a suitable measure of LD for mtDNA based on certain statistical criteria, such as for maximum values of r^2^ to be achieved all alleles must have equal frequency (Jorde and Bamshad 2000; Kumar *et al.* 2000). In contrast, it has been suggested that the r^2^ measure of LD is preferable over D′ for synonymous mutations in human mtDNA because synonymous sites in human mtDNA are described well by the finite sites model (Piganeau and Eyre-Walker 2004), a model for which r^2^ is more suited (McVean *et al.* 2002). Simulation studies have generally not been able to differentiate the relative efficacies of using D′ or r^2^ (Bruen *et al.* 2006; Meunier and Eyre-Walker 2001). Like Bruen *et al.* (2006), we found that r^2^ is slightly less powerful under population growth, although in another study White and Gemmell (2009) give an example in which a test based on r^2^ detected recombination in human mtDNA when D′ did not—the only case in which we know recombination has occurred. It has been suggested that hypervariable mutation leading to recurrent mutation would cause a reduction in detection using D′ but not r^2^ (Meunier and Eyre-Walker 2001), and because this parameter is not included in our simulations here (whereas other evolutionary factors are), hypervariability could explain the discrepancy between the results presented here and those of our earlier analyses of empirical and simulated data. It would be interesting to explore further the effect of recurrent mutation on these tests.

It is perhaps the scenario of mutation rate heterogeneity and population growth that is most relevant for human mtDNA, as these phenomena are believed to be major factors of human mtDNA evolution and recent demographic history (Pluzhnikov *et al.* 2002; Yang 1996). As only minor differences are seen when mutation rate heterogeneity alone is included in models of sequence evolution, it is probable that population growth, and the subsequent reduction in sequence diversity, is the major contributor to the marked reduction in efficiency for tests under a slightly more complex scenario. Thus, it appears that the parameters under which human mtDNA is evolving have a substantial impact on the performance of these indirect tests of recombination.

Ignoring the Homoplasy Test due to its inflated false-positive rate, and considering the discrepancies in the LD D′ test with previous studies (White and Gemmell 2009), it is the incompatibility tests that are most robust to the parameters of sequence evolution implemented here, while maintaining low false-positive rates. Because the PHI test was unable to detect recombination in empirical data (White and Gemmell 2009), the NSS test may be the most suited of the tests included here to detect recombination in human mtDNA. The LD r^2^ test may also be useful.

The focus of this study, however, was not to provide estimates of the power of these tests to detect recombination, but rather to test how the parameters under which human mtDNA evolve might affect the efficacy of indirect tests of recombination. In general, the six approaches tested, all of which have been widely applied for the detection of recombination in sequence data, perform poorly. We demonstrate that under a realistic rate of recombination for human mtDNA, the performance of most indirect tests is reduced at least twofold when population growth and mutation rate heterogeneity are considered compared with a scenario when these factors are absent. Further, it is apparent that error rates vary dramatically across tests and that relying on a universal nominal *P* value of 0.05 to demonstrate statistical significance is simply not appropriate. As the evolution of human mtDNA is likely to be more complex than we simulated here, we strongly recommend further development of indirect tests of recombination for accurate detection in human mtDNA. At the very least, we suggest that simulations be run to explore the limitations of tests as part of empirical investigations.

If we were to reinvestigate past efforts to detect recombination in human populations, considering the results of this study, conclusions may need to be reviewed. Studies thus far have used several indirect tests, including those that we have shown to be unsuitable for human mtDNA, and evidence has generally been negative or inconclusive (Elson *et al.* 2001; Herrnstadt *et al.* 2002; Ingman *et al.* 2000; Jorde and Bamshad 2000; Piganeau and Eyre-Walker 2004). By removing nonsuitable tests from studies, the landscape of evidence for no recombination in human populations changes. For example, in the most comprehensive screen for recombination in human mtDNA using indirect means to date, Piganeau and Eyre-Walker (2004) used five tests on six population data sets and obtained twenty-eight *P* values. Of these 28, seven (25%) were statistically significant. However, if we remove *P* values obtained using LD *vs.* distance using D′ and the Max χ^2^ test (the implementation of Max χ^2^ in their study did not account for expected χ^2^values of less than 2), seven of 17 *P* values achieve statistical significance, or around 41%.

Recombination in human mtDNA, if it is occurring, could not only have a dramatic impact on our understanding of disease etiology and transmission but also on numerous evolutionary inferences. Even small amounts could adversely compound evolutionary dating and bias phylogenetic analyses (Schierup and Hein 2000a,b; White *et al.* 2008). Recombination can lead to false discovery of positive selection using the dN/dS ratio, leading to erroneous interpretation of the impact of selection across genomes (Anisimova *et al.* 2003; Arenas and Posada 2010a). Further, recombination has a direct and confounding effect on ancestral sequence reconstruction (Arenas and Posada 2010b). It is important, therefore, to obtain accurate estimates of mitochondrial recombination at the population level. Although attempts have been made, indirect evidence for mtDNA recombination in humans has been unconvincing thus far, despite compelling direct evidence. Here, we show that even if recombination in human mtDNA were to occur, its detection is not guaranteed using the currently available tests, and if we were to project from the trends presented here we would expect values of rho to need to be around three to four times that seen *in vivo* (*i.e.*, around 20), for rates of recombination detection to be in the 90% range for the tests reviewed here. This leads to the possibility that recombination could be occurring in human mtDNA at the population level, but that these levels are below current detection thresholds. This study is timely, as the advance of the new wave of sequencing technology will lead to many further opportunities to screen human populations for the presence of mitochondrial recombination. We foresee results from our work leading to improved accuracy of detection of human mitochondrial recombination frequency at the population level.

## Supplementary Material

Supporting Information

## References

[bib1] Anisimova M. Nielsen R. Yang Z. H., 2003 Effect of recombination on the accuracy of the likelihood method for detecting positive selection at amino acid sites. Genetics 164: 1229–1236 12871927 10.1093/genetics/164.3.1229 PMC1462615

[bib2] Arenas M. Posada D., 2007 Recodon: coalescent simulation of coding DNA sequences with recombination, migration and demography. BMC Bioinformatics 8: 458 18028540 10.1186/1471-2105-8-458 PMC2206059

[bib3] Arenas M. Posada D., 2010 a Coalescent simulation of intracodon recombination. Genetics 184: 429–437 19933876 10.1534/genetics.109.109736 PMC2828723

[bib4] Arenas M. Posada D., 2010 b The effect of recombination on the reconstruction of ancestral sequences. Genetics 184: 1133–1139 20124027 10.1534/genetics.109.113423 PMC2865913

[bib5] Awadalla P. Eyre-Walker A. Smith J. M., 1999 Linkage disequilibrium and recombination in hominid mitochondrial DNA. Science 286: 2524–2525 10617471 10.1126/science.286.5449.2524

[bib6] Brown C. J. Garner E. C. Dunker A. K. Joyce P., 2001 The power to detect recombination using the coalescent. Mol. Biol. Evol. 18: 1421–1424 11420381 10.1093/oxfordjournals.molbev.a003927

[bib7] Bruen T. C. Philippe H. Bryant D., 2006 A simple and robust statistical test for detecting the presence of recombination. Genetics 172: 2665–2681 16489234 10.1534/genetics.105.048975 PMC1456386

[bib8] D’Aurelio M. Gajewski C. D. Lin M. T. Mauck W. M. Shao L. Z., 2004 Heterologous mitochondrial DNA recombination in human cells. Hum. Mol. Genet. 13: 3171–3179 15496432 10.1093/hmg/ddh326

[bib9] Elson J. L. Andrews R. M. Chinnery P. F. Lightowlers R. N. Turnbull D. M., 2001 Analysis of European mtDNAs for recombination. Am. J. Hum. Genet. 68: 145–153 11115380 10.1086/316938 PMC1234908

[bib10] Eyre-Walker A. Smith N. H. Smith J. M., 1999 How clonal are human mitochondria? Proc. R. Soc. Lond. B Biol. Sci. 266: 477–483 10.1098/rspb.1999.0662 PMC1689787 10189711

[bib11] Fontaine K. M. Cooley J. R. Simon C., 2007 Evidence for paternal leakage in hybrid periodical cicadas (Hemiptera: Magicicada spp.). PLoS ONE 2: e892 17849021 10.1371/journal.pone.0000892 PMC1963320

[bib12] Gyllensten U. Wharton D. Josefsson A. Wilson A. C., 1991 Paternal inheritance of mitochondrial-dna in mice. Nature 352: 255–257 1857422 10.1038/352255a0

[bib13] Hagelberg E. Goldman N. Lio P. Whelan S. Schiefenhovel W., 1999 Evidence for mitochondrial DNA recombination in a human population of island Melanesia [correction in Proc R Soc Lond B Biol Sci 2000;267:1595−1596.]. Proc. R. Soc. Lond. B Biol. Sci. 266: 485–492 10.1098/rspb.1999.0663 PMC1689791 10189712

[bib14] Herrnstadt C. Elson J. L. Fahy E. Preston G. Turnbull D. M., 2002 Reduced-median-network analysis of complete mitochondrial DNA coding-region sequences for the major African, Asian, and European haplogroups. Am. J. Hum. Genet. 70: 1152–1171 11938495 10.1086/339933 PMC447592

[bib15] Ingman M. Kaessmann H. Paabo S. Gyllensten U., 2000 Mitochondrial genome variation and the origin of modern humans. Nature 408: 708–713 11130070 10.1038/35047064

[bib16] Innan H. Nordborg M., 2002 Recombination or mutational hot spots in human mtDNA? Mol. Biol. Evol. 19: 1122–1127 12082131 10.1093/oxfordjournals.molbev.a004170

[bib17] Ivanov P. L. Wadhams M. J. Roby R. K. Holland M. M. Weedn V. W., 1996 Mitochondrial DNA sequence heteroplasmy in the Grand Duke of Russia Georgij Romanov establishes the authenticity of the remains of TsarNicholas II. Nat. Genet. 12: 417–420 8630496 10.1038/ng0496-417

[bib18] Jakobsen I. B. Easteal S., 1996 A program for calculating and displaying compatibility matrices as an aid in determining reticulate evolution in molecular sequences. Comput. Appl. Biosci. 12: 291–295 8902355 10.1093/bioinformatics/12.4.291

[bib19] Jorde L. B. Bamshad M., 2000 Questioning evidence for recombination in human mitochondrial DNA. Science 288: 1931 10877699

[bib20] Kivisild T. Villems R., 2000 Questioning evidence for recombination in human mitochondrial DNA. Science 288: 1931a 10877700

[bib21] Kraytsberg Y. Schwartz M. Brown T. A. Ebralidse K. Kunz W. S., 2004 Recombination of human mitochondrial DNA. Science 304: 981 15143273 10.1126/science.1096342

[bib22] Kuhner M. K., 2006 LAMARC 2.0: maximum likelihood and Bayesian estimation of population parameters. Bioinformatics 22: 768–770 16410317 10.1093/bioinformatics/btk051

[bib23] Kumar S. Hedrick P. Dowling T., 2000 Questioning evidence for recombination in human mitochondrial DNA. Science 288: 1931 10877701

[bib24] Lakshmipathy U. Campbell C., 1999 The human DNA ligase III gene encodes nuclear and mitochondrial proteins. Mol. Cell. Biol. 19: 3869–3876 10207110 10.1128/mcb.19.5.3869 PMC84244

[bib25] McVean G. Awadalla P. Fearnhead P., 2002 A coalescent-based method for detecting and estimating recombination from gene sequences. Genetics 160: 1231–1241 11901136 10.1093/genetics/160.3.1231 PMC1462015

[bib26] Meunier J. Eyre-Walker A., 2001 The correlation between linkage disequilibrium and distance: Implications for recombination in hominid mitochondria. Mol. Biol. Evol. 18: 2132–2135 11606711 10.1093/oxfordjournals.molbev.a003756

[bib27] Mishmar D. Ruiz-Pesini E. Golik P. Macaulay V. Clark A. G., 2003 Natural selection shaped regional mtDNA variation in humans. Proc. Natl. Acad. Sci. USA 100: 171–176 12509511 10.1073/pnas.0136972100 PMC140917

[bib28] Piganeau G. Eyre-Walker A., 2004 A reanalysis of the indirect evidence for recombination in human mitochondrial DNA. Heredity 92: 282–288 14747833 10.1038/sj.hdy.6800413

[bib29] Piganeau G. Gardner M. Eyre-Walker A., 2004 A broad survey of recombination in animal mitochondria. Mol. Biol. Evol. 21: 2319–2325 15342796 10.1093/molbev/msh244

[bib30] Pluzhnikov A. Di Rienzo A. Hudson R. R., 2002 Inferences about human demography based on multilocus analyses of noncoding sequences. Genetics 161: 1209–1218 12136023 10.1093/genetics/161.3.1209 PMC1462170

[bib31] Posada D. Crandall K. A., 2001 Evaluation of methods for detecting recombination from DNA sequences: computer simulations. Proc. Natl. Acad. Sci. USA 98: 13757–13762 11717435 10.1073/pnas.241370698 PMC61114

[bib32] Schierup M. H. Hein J., 2000 a Consequences of recombination on traditional phylogenetic analysis. Genetics 156: 879–891 11014833 10.1093/genetics/156.2.879 PMC1461297

[bib33] Schierup M. H. Hein J., 2000 b Recombination and the molecular clock. Mol. Biol. Evol. 17: 1578–1579 11018163 10.1093/oxfordjournals.molbev.a026256

[bib34] Schwartz M. Vissing J., 2002 Paternal inheritance of mitochondrial mtDNA. N. Engl. J. Med. 347: 576–580 12192017 10.1056/NEJMoa020350

[bib35] Sherengul W. Kondo R. Matsuura E. T., 2006 Analysis of paternal transmission of mitochondrial DNA in Drosophila. Genes Genet. Syst. 81: 399–404 17283385 10.1266/ggs.81.399

[bib36] Smith J. M., 1992 Analyzing the mosaic structure of genes. J. Mol. Evol. 34: 126–129 1556748 10.1007/BF00182389

[bib37] Smith J. M. Smith N. H., 1998 Detecting recombination from gene trees. Mol. Biol. Evol. 15: 590–599 9580989 10.1093/oxfordjournals.molbev.a025960

[bib38] Sun S. Evans B. J. Golding G. B., 2011 “Patchy-Tachy” leads to false positives for recombination. Mol. Biol. Evol. 28: 2549–2559 21498600 10.1093/molbev/msr076

[bib39] Thyagarajan B. Padua R. A. Campbell C., 1996 Mammalian mitochondria possess homologous DNA recombination activity. J. Biol. Chem. 271: 27536–27543 8910339 10.1074/jbc.271.44.27536

[bib40] Tsaousis A. D. Martin D. P. Ladoukakis E. D. Posada D. Zouros E., 2005 Widespread recombination in published animal mtDNA sequences. Mol. Biol. Evol. 22: 925–933 15647518 10.1093/molbev/msi084

[bib41] White D. J. Gemmell N. J., 2009 Can indirect tests detect a known recombination event in human mtDNA? Mol. Biol. Evol. 26: 1435–1439 19369597 10.1093/molbev/msp073

[bib42] White D. J. Wolff J. N. Pierson M. Gemmell N. J., 2008 Revealing the hidden complexities of mtDNA inheritance. Mol. Ecol. 17: 4925–4942 19120984 10.1111/j.1365-294X.2008.03982.x

[bib43] Wiuf C. Christensen T. Hein J., 2001 A simulation study of the reliability of recombination detection methods. Mol. Biol. Evol. 18: 1929–1939 11557798 10.1093/oxfordjournals.molbev.a003733

[bib44] Yang Z. H., 1996 Among-site rate variation and its impact on phylogenetic analyses. Trends Ecol. Evol. 11: 367–372 21237881 10.1016/0169-5347(96)10041-0

[bib45] Zhao X. Li N. Guo W. Hu X. Liu Z., 2004 Further evidence for paternal inheritance of mitochondrial DNA in the sheep (Ovis aries). Heredity 93: 399–403 15266295 10.1038/sj.hdy.6800516

[bib46] Zsurka G. Hampel K. G. Kudina T. Kornblum C. Kraytsberg Y., 2007 Inheritance of mitochondrial DNA recombinants in double-heteroplasmic families: Potential implications for phylogenetic analysis. Am. J. Hum. Genet. 80: 298–305 17236134 10.1086/511282 PMC1785346

[bib47] Zsurka G. Kraytsberg Y. Kudina T. Kornblum C. Elger C. E., 2005 Recombination of mitochondrial DNA in skeletal muscle of individuals with multiple mitochondrial DNA heteroplasmy. Nat. Genet. 37: 873–877 16025113 10.1038/ng1606 PMC1444756

