# Supplementary material for: How Good Are Indirect Tests at Detecting Recombination in Human mtDNA?
Source: G3 (Bethesda). 2013 Jul 1;3(7):1095–104. doi: 10.1534/g3.113.006510 (PMC3704238; doi:10.1534/g3.113.006510)
Supplement: Supporting Information [file supp_3_7_1095__index.html]

How Good Are Indirect Tests at Detecting Recombination in Human mtDNA? — Supporting Information 

# How Good Are Indirect Tests at Detecting Recombination in Human mtDNA?

## Supporting Information for White, Bryant, and Gemmell, 2013

**Files in this Data Supplement:**

- Supporting Information - Files S1-S2 and Table S1 (PDF, 350 KB)
- Table S1 - Validation of the C translation of the Homoplasy Test (PDF, 208 KB)
- File S1 - Detailed Description of the Indirect Tests of Recombination (PDF, 267 KB)
- File S2 - Simulated sequence data (.gz, 12 MB)
