# Supplementary material for: How Good Are Indirect Tests at Detecting Recombination in Human mtDNA?
Source: G3 (Bethesda). 2013 Jul 1;3(7):1095–104. doi: 10.1534/g3.113.006510 (PMC3704238; doi:10.1534/g3.113.006510)
Supplement: Supporting Information [file supp_g3.113.006510_TableS1.pdf]

Table S1 Validation of the C translation of the Homoplasy Test

	QB				C					QB				C			
**File**	Inf	TrH	Se	p<0.05?	Inf	TrH	Se	p<0.05?	**File**	Inf	TrH	Se	p<0.05?	Inf	TrH	Se	p<0.05?
**0_1**	14	1	1370	No	14	1	1370	No	**15_1**	21	15	1370	Yes	21	15	1370	Yes
**0_4**	17	0	1370	No	17	0	1370	No	**15_2**	8	3	1370	Yes	8	3	1370	Yes
**0_5**	23	2	1370	Yes	23	2	1370	Yes	**15_3**	19	16	1370	Yes	19	16	1370	Yes
**0_6**	19	1	1370	No	19	1	1370	Yes	**15_4**	12	4	1370	Yes	12	4	1370	Yes
**0_7**	23	0	1370	No	23	0	1370	No	**15_5**	21	11	1370	Yes	21	11	1370	Yes

Whether significance at a p value of 0.05 was achieved for five files with zero recombination (ρ=0) and five files with an extreme level of recombination (ρ=15) is shown. QB: QuickBasic, C: C translation, Inf: informative sites, TrH: true homoplasies, Se: effective sites.
