# Supplementary material for: How Good Are Indirect Tests at Detecting Recombination in Human mtDNA?
Source: G3 (Bethesda). 2013 Jul 1;3(7):1095–104. doi: 10.1534/g3.113.006510 (PMC3704238; doi:10.1534/g3.113.006510)
Supplement: Supporting Information [file supp_g3.113.006510_FileS1.pdf]

File S1

Detailed Description of the Indirect Tests of Recombination

LD vs distance

In the presence of recombination, the strength of linkage disequilibrium between two alleles should decrease with physical distance. A significant negative correlation could, theoretically, indicate recombination. There are several measures of LD, and two have been used for assessing recombination in mtDNA: r^2 and D' (Hill and Robertson 1968; Lewontin 1964). Both are dependent on allele frequencies but with slightly different properties. They are described by

D' = D / Dmax (Lewontin 1964), and

$r^2 = D^2 / p\text{A}p\text{a}p\text{B}p\text{b}$ (Hill and Robertson 1968),

where D is the linkage disequilibrium coefficient, D = *p*AB – *p*A*p*B; A, B, a and b are alleles; AB is a haplotype composed of alleles A and B; and *p* is population frequency. For both measures of LD Pearson's correlation coefficient was used, and the statistical significance of the correlation was estimated after 1000 random permutations of the data using a Mantel test, all implemented in the stand-alone version of RecombiTEST (Piganeau *et al.* 2004).

Homoplasy Test

If more homoplasies (co-occurrence of a polymorphism on separate branches of a phylogenetic tree) occur in a most parsimonious tree than expected by recurrent mutation under a model of clonal inheritance, then recombination may be the most likely explanation (Smith and Smith 1998). This was tested using the Homoplasy Test (Smith and Smith 1998) and implemented in the Linux operating system using a C translation of the original QBasic version, kindly provided by David Posada (University of Vigo). To simulate the process of synonymous site selection, a step recommended by the authors to control for the compounding effects of selection on recombination detection, a second file was generated for analysis, using every third base pair of the 6854bp-long simulated sequence.

Max Chi Squared

The Max Chi Squared method compares the arrangement of segregating sites between 2 sequences either side of a putative recombination break point, with all other sequences in the alignment (Smith 1992). A sliding window of sequence to analyse was set to the number of polymorphic sites divided by 1.5, and the window moved along the alignment one base pair at a time. Significance was estimated as the proportion of 1000 permuted test scores, calculated after randomly positioning the informative sites, below the observed test score.

Neighbour Similarity Score

The NSS describes the extent of clustering of compatibilities (either compatible or incompatible) of adjacent informative sites in a sequence alignment (Jakobsen and Easteal 1996). Two sites are said to be compatible only if their history includes no recurrent or convergent mutation, otherwise they are incompatible. Higher NSS values than expected by chance can be explained by recurrent mutation, gene conversion or recombination. Significance of the observed NSS is achieved by randomly permuting the order of informative sites 1000 times, and determining the fraction of random scores that are at least as high as the observed data.

Pairwise Homoplasy Index

The PHI measures the mean refined incompatibility score between sites within a window of sequence of preset length, and reflects the minimum number of homoplasies on any tree required to describe the genealogical history of a pair of sites (Bruen *et al.* 2006). Compatibility is negatively correlated with recombination. If recombination is responsible for homoplasies, PHI scores should be lower than if recurrent mutation is responsible, as recurrent mutation is not correlated with physical distance. An estimate of the statistical significance of the PHI was achieved by randomly permuting site positions in the alignment (simulating no recombination) 1000 times, and calculating the proportion of times the permuted PHI score is less than or equal to the observed score.

C versions of Max Chi Squared, NSS and PHI was run in linux and can be downloaded from http://www.maths.otago.ac.nz/~dbryant/software.html.

References

Bruen, T. C., H. Philippe and D. Bryant, 2006 A simple and robust statistical test for detecting the presence of recombination. Genetics **172:** 2665-2681.

Hill, W. G., and A. Robertson, 1968 Linkage disequilibrium in finite populations. Theor Appl Genet **38:** 226-231.

Jakobsen, I. B., and S. Easteal, 1996 A program for calculating and displaying compatibility matrices as an aid in determining reticulate evolution in molecular sequences. Comput Appl Biosci **12:** 291-295.

Lewontin, R. C., 1964 Interaction of Selection + Linkage .I. General Considerations - Heterotic Models. Genetics **49:** 49-67.

Piganeau, G., M. Gardner and A. Eyre-Walker, 2004 A broad survey of recombination in animal mitochondria. Mol Biol Evol **21:** 2319-2325.

Smith, J. M., 1992 Analyzing the Mosaic Structure of Genes. Journal of Molecular Evolution **34:** 126-129.

Smith, J. M., and N. H. Smith, 1998 Detecting recombination from gene trees. Mol Biol Evol **15:** 590-599.
